# Sampling globally and locally correct RNA 3D structures using Ernwin, SPQR and experimental SAXS data

**DOI:** 10.1093/nar/gkae602

**Published:** 2024-07-18

**Authors:** Bernhard C Thiel, Giovanni Bussi, Simón Poblete, Ivo L Hofacker

**Affiliations:** Department of Theoretical Chemistry, University of Vienna, Währinger Strasse 17, Vienna 1090, Austria; Scuola Internazionale Superiore di Studi Avanzati, SISSA, via Bonomea 265, Trieste 34136, Italy; Centro BASAL Ciencia & Vida, Avenida del Valle Norte 725, Santiago 8580702, Chile; Facultad de Ingeniería, Arquitectura y Diseño, Universidad San Sebastián, Bellavista 7, Santiago 8420524, Chile; Department of Theoretical Chemistry, University of Vienna, Währinger Strasse 17, Vienna 1090, Austria; Research group Bioinformatics and Computational Biology, Faculty of Computer Science, University of Vienna, Vienna 1090, Austria

## Abstract

The determination of the three-dimensional structure of large RNA macromolecules in solution is a challenging task that often requires the use of several experimental and computational techniques. Small-angle X-ray scattering can provide insight into some geometrical properties of the probed molecule, but this data must be properly interpreted in order to generate a three-dimensional model. Here, we propose a multiscale pipeline which introduces SAXS data into modelling the global shape of RNA in solution, which can be hierarchically refined until reaching atomistic precision in explicit solvent. The low-resolution helix model (Ernwin) deals with the exploration of the huge conformational space making use of the SAXS data, while a nucleotide-level model (SPQR) removes clashes and disentangles the proposed structures, leading the structure to an all-atom representation in explicit water. We apply the procedure on four different known pdb structures up to 159 nucleotides with promising results. Additionally, we predict an all-atom structure for the *Plasmodium falceparum* signal recognition particle ALU RNA based on SAXS data deposited in the SASBDB, which has an alternate conformation and better fit to the SAXS data than the previously published structure based on the same data but other modelling methods.

## Graphical Abstract

**Graphical Abstract:**
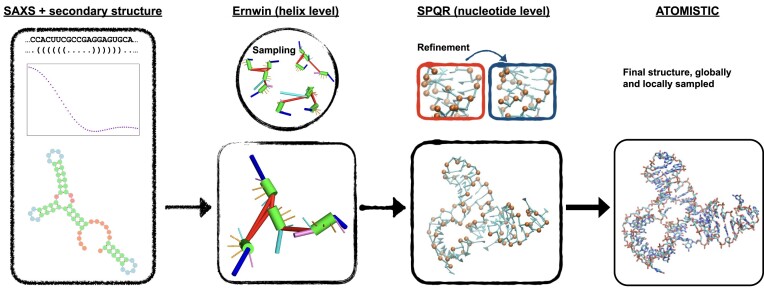

## Introduction

The incorporation of experimental data into the structural modelling of nucleic acids is an increasingly common approach, which helps to scale down the sea of possible structures that computer algorithms can propose (1). Among these experimental techniques, small-angle X-ray scattering (SAXS) is a handy tool for providing insight into the global shape and size of macromolecules in solution (2,3), which can be employed to refine models or to guide molecular dynamics (MD) simulations. The information provided by the SAXS curve is, however, a 1D intensity profile of limited resolution, which opens the questions of how to interpret and integrate this data into an all-atom model. Although in principle the scattering intensities can be calculated exactly from the atomic coordinates, the effect of the hydration shell is not known in a closed form, and it is usually taken into account through different approximations in diverse tools. For example, an implicit water model is used in PLUMED (4) and in the original version of CRYSOL (5), but later versions of this software employ dummy water beads to model the hydration shell (6), in the same spirit as Fast-SAXS-pro (7). More complex methods, such as WAXSiS (8) and Capriqorn (9) take into explicit consideration the solvent effects, by analyzing the SAXS contribution of an entire set of MD-generated trajectories, and subtracting the solvent effects obtained from an independent simulation of the buffer. In all these approaches, there is a trade-off between speed and accuracy (10), which has to be taken into account when dealing with large RNA structures.

There is also a variety of ways for building molecular models based on SAXS profiles. A common approach is to generate a huge number of structures and to afterwards select those with the best match between the predicted SAXS profile and the experimental data (11). SAXS intensities have also been used to reweight or sample from MD simulations (12,13). Other scattering profiles have been employed in a similar fashion (14). These procedures, however, become less efficient with increasing system size. A more sophisticated treatment uses SAXS data during sampling. This idea has been applied to MD simulations schemes, either in the form in guided sampling (15) or aiming at force-field refinement (16). By assembling fragments based on the predicted secondary structure (similar to the ones we use in the present work) and calling CRYSOL in every sampling step, Gajda *et al.* (17) managed to generate good predictions for structures of up to 70 nucleotides. Similarly, Dzananovic *et al.* (18) applied such an approach to a viral three-way junction of roughly 50 nts length. Their tool, called RNA Masonry (19), also performs fragment assembly but uses the SimRNA (20) energy function in addition to CRYSOL for the evaluation of every structure. For larger RNA molecules, the program RS3D (21) can be used, employing a coarse-grained (CG) representation. In the same vein, the CG model HiRe-RNA has also been extended to guide its simulations with an energy function dependent on SAXS intensities on structures up to 77 nucleotides (22).

Here, we present an approach that is suitable for large RNA structures due to the use of a coarse-grained representation of the fragments based on the secondary structure, which is hierarchically refined going through a higher resolution coarse-grained model until atomistic resolution. We incorporate SAXS data via the pair distance distribution function at every sampling step, which is more intuitively accessible to non-crystallographers than the intensity distribution in reciprocal space.

In addition to the possibility to search for a single best structure, we also describe an ensemble-based approach, which optimizes the SAXS profile of the ensemble as opposed to optimizing an individual structure. This way, we account for the intrinsic flexibility of large RNA molecules in solution. In the refinement step, we propose a method for removing clashes and fixing broken bonds, and pay special attention to the entanglements, both in their detection and removal, and in the possibility of forming tertiary contacts.

The pipeline starts from the secondary structure and SAXS data, which is used for defining a helix-based representation in an improved version of the Ernwin model (23) for exploring and adjusting the global shape of the RNA molecule. Afterwards, the best results are refined by means of the SPlit and conQueR (SPQR) model (24), a nucleotide-level resolution coarse-grained description. Finally, the structures are backmapped into an all-atom representation for further MD simulation in explicit solvent. This multiscale approach integrates the strengths of each representation in a way that it resembles the hierarchical folding of RNA.

## Materials and methods

**Figure 1.**
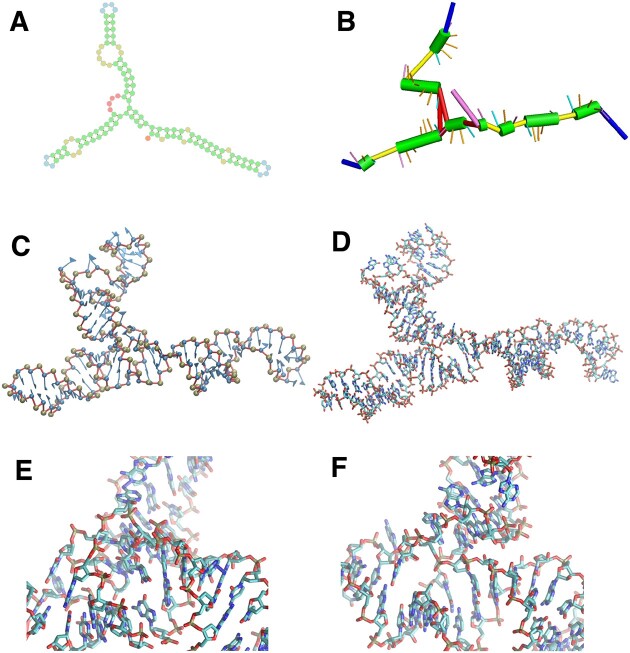
Representation of an RNA structure under different levels of resolution: (**A**) Secondary structure, (**B**) Ernwin, helix-based, (**C**) SPQR, nucleotide-based and (**D**) atomistic. A typical junction with clashes is shown in (**E**), while (**F**) is the same region after the refinement presented here.

In this section we will first briefly describe Ernwin, and introduce the new features we have added to it and explain the incorporation of SAXS data into its energy function. Later, after a brief introduction of the SPQR model, its capabilities used into the refinement procedure will be exposed, while the subsequent MD refinement will be explained in the Supplementary Data. Finally, we present the way our pipeline is assembled and tested. Overall, the different levels of resolution of the pipeline are illustrated in Figure 1, with an example of a three-way junction where several clashes are presented and removed after the refinement procedure.

### Coarse-grained structure prediction by Ernwin

Our in-house RNA structure prediction tool Ernwin (23) assembles RNA 3D structures from fragments of known crystal structures, which are defined via the secondary structure elements (stems, interior loops, etc.). A rigid coarse-grained representation of these elements is used during sampling.

As described previously (23), stems are parametrized by their length and the orientation of the minor groove along the helix. By assuming that each base pair of the helix contributes equally to the helix rotation, we can calculate ‘virtual’ residue and atom positions, which are in good agreement with the true positions. These virtual residue positions can be calculated on the fly and do not have to be stored. More interesting for sampling than stems are the internal loops which connect the stems and introduce angles and offsets between them. Interior loops and multi-loop segments are parametrized by the distance between and relative orientation of the adjacent stems as well as the relative location of the stems’ minor grooves.

#### Ernwin energy terms

We have previously shown how the reference ratio method can be applied to the sampling of RNA structures (23): For a given measure of interest, such as the radius of gyration, we supply a target distribution of values taken from solved RNA structures. During sampling we compare the distribution of this measure in our sampled structures to this target distribution and calculate a pseudo-energy based on their difference. We use this pseudo-energy in a Metropolis–Rosenbluth–Hastings like accept-reject step and in this way fit the distribution of the measure of interest over the sampled ensemble to the target distribution, irrespective of the distribution this measure would have without the use of an energy.

During the last years, we have retrained the energy potentials on the representative set of RNA 3D structures (25). Furthermore, we have adapted the contributions of long range interactions (loop-loop interaction and A-Minor interaction) to the energy function to better suit longer and more extended RNA molecules. See Supplementary Data Section 1.1 for more details. In addition, Ernwin can make use of known RNA 3D motifs, i.e. loops with non-canonical base pairs (26,27); however, this feature is not used in the present paper.

For fitting SAXS data, we replaced the radius of gyration energy by a pair distance distribution energy. See section ‘Fitting the ensemble to the SAXS derived PDD’ below for details.

#### Handling of multiloops

A three-way junction has three single-stranded regions, but is fully defined after sampling only two single-strand fragments, as there are no degrees of freedom left for the third fragment. In such situations, Ernwin originally only restricted the length in 3D space that is spanned by the last segment (which we will call *‘broken’ multiloop segment*) and did not perform any sampling. Unfortunately, this approach led to a bias in the distribution of sampled multiloop topologies and generation of unrealistic conformations (see Results).

Here, we explore new ways of assigning a fragment to the *broken multiloop segment* and accept or reject the multiloop conformation based on its fit. We choose to calculate this fit based on the deviation between the first stem of the multiloop and the way we would place this exact same stem after going around the junction according to the sampled fragments. See the Supplementary Data Section 1.2 and Supplementary Figure S1 for details on how we assign fragments to the *broken multiloop segment* and how we benchmark them.

#### Move sets

The most straightforward way to go from one structure to the next during sampling is by changing a single fragment. Due to the fragment based approach, even a single fragment change can potentially change completely the overall conformation, e.g. when we change the angle between two arms of an RNA. These big steps help with quickly exploring the conformational space. However, as the individual parts of the RNA molecule are not independent, in particular if they are linked in a multiloop, big changes to certain fragments have a high chance of introducing clashes or breaking the multiloop constraints, especially in large molecules with complex secondary structure. As Ernwin rejects structures with clashes, this type of sampling is often inefficient.

For the present version of Ernwin we have added and tested five alternative move types, which are all based on the idea of moving more than one fragment at a time. Most suitable for simple junctions is to move two or three connected fragments of the same multiloop at a time. For very complex structures we implemented a move type that consists of an exchange of a single fragment followed by a relaxation step based on the clash and multiloop constraints. The other move types and the way we benchmark them are described in the Supplementary Data Section 1.3.

#### Fitting the ensemble to the SAXS derived PDD

The observed SAXS pattern in reciprocal space can be converted to the pair distance distribution function (PDD) in real space via an indirect Fourier transform, and creating this pair distance distribution function is a standard part of SAXS analysis using tools like GNOM (28).

We interpret the PDD as a distribution of distances between the atomic centers. Although this approximation neglects the effects of the hydration shell, we have found that it is sufficient to produce good simulation results. Ernwin has the option to use one point (C1’) or three points (base, sugar and backbone) per residue for calculating the pair distance distribution function. While one point per residue was sufficient for simulating the *braveheart* lncRNA (636 nts) (29), we used 3 points in this publication, as we were dealing with shorter RNA molecules.

We implemented two energy functions which act on the PDD in different ways: Either as a potential on the area between the two PDD-curves, or by applying the reference ratio method to each histogram bin of the PDD individually. The latter approach is more powerful, as it operates on the ensemble of sampled structures instead of just a single structure. See the Supplementary Data Section 1.4 and Supplementary Figure S2 for implementation details of these approaches.

### Nucleotide-level refinement

We employ the SPQR model to refine the reconstructed decoys at nucleotide level. The model represents each nucleotide by a phosphate bead and the nucleoside by an anisotropic particle. The interactions allow forming stacking, canonical and non-canonical base pairs, and base-phosphate pairs, while the sugar pucker and glycosidic bond angle states are additional degrees of freedom of each nucleotide (24), and can be allowed to change dynamically along a simulation.

In the present framework, the structures with broken bonds and clashes, which have an undefined SPQR energy, can be treated with special energy terms which push the involved particles towards a finite-energy region. The definition of the energy terms and parameters are described in the Supplementary Data Section 2.1. This relaxation is, however, not enough to guarantee the aptness of the model, since it is common that during the assembly steps the secondary structure elements become entangled. SPQR detects and attempts to remove these artifacts, and later proceeds to perform a search for the possible tertiary contacts proposed by Ernwin. These procedures are described below in more detail.

#### Link detection and removal

**Figure 2.**
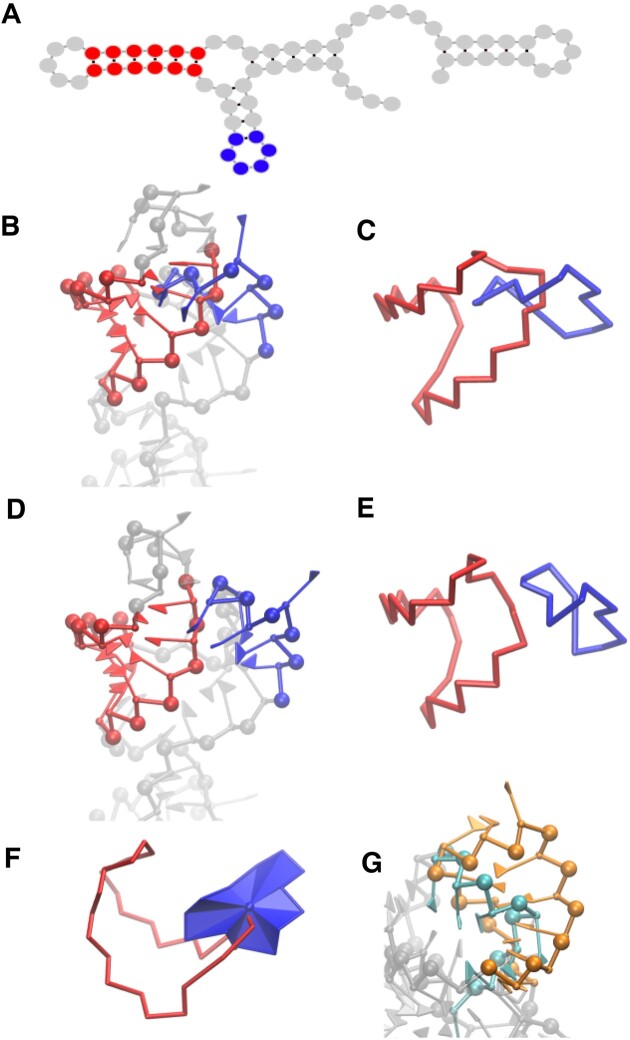
Examples of links for identification and removal (**A**) Secondary structure, (**B**) a hairpin and a stem linked, (**C**) ring representation of the link, (**D**) after clash and link removal and (**E**) ring representation. (**F**) Top view of the link, with triangulated surface of hairpin and (**G**) interpenetrated hairpin and stem, not detected with Gauss integral method.

To detect and fix entangled secondary structure elements such as hairpins, stems and internal loops, we represent each of them as a closed ring, as depicted in Figure 2A–E. In practice, these rings are constituted by the segments that join the positions of consecutive phosphate, sugar beads and closing base pairs. We then apply two approaches, previously reported in the literature (30,31), which are combined for efficiency and robustness.

The first approach makes use of a known way in knot theory of checking whether two rings are linked by evaluated the linking number *L*, defined by the Gauss linking integral:


(1) \begin{equation*} L(R_i,R_j)=\frac{1}{4\pi }\oint _{R_i}\oint _{R_j}\frac{({\bf r}_{i}-{\bf r}_{j})}{|{\bf r}_i-{\bf r}_j|^3} \cdot (d{\bf r}_i \times d {\bf r}_j), \end{equation*}

where the vectors **r**_*i*_ and **r**_*j*_ go along the rings *R*_*i*_ and *R*_*j*_ respectively. *L* is the number of times that one ring winds around the other one, which is zero if the rings are not linked. In our case, we evaluate the integrals numerically along segments defining our ring elements. This approach has already been used in the study of the topology of lasso-type proteins with disulfide bonds (30) and to characterize the topology of proteins (32).

The second approach, which has also been tested in proteins (31) and nucleic acids (33), consists in evaluating the number of times that the segments which compose a ring pierce the surface enclosed by another ring. In the present case, we consider such a surface as a set of adjacent triangles, formed by two consecutive vertices and the center of mass of the ring; a similar but simpler implementation than the one reported in (31). Such a representation is illustrated in Figure 2F.

The Gauss integral procedure is in general slower, but it allows to include more complex objects in the analysis, such as three- and four-way junctions, which can also be defined as closed rings and can be difficult to define as a set of triangles due to their geometry. The piercing method, on the other hand, permits the detection of rings crossed by elements such as internal loops which can not be included into a closed ring or short backbone segments between consecutive stems. Most of the occurrences detected by this approach correspond exactly to the links detected by the Gauss integral evaluation, while false positives usually stand for the interpenetration of hairpins and stems of spurious origin, which often involve a large number of clashes in an intricate manner as shown in Figure 2G. After the links are detected, a repulsive energy term is imposed between the nucleotides involved and specific virtual sites defined on each ring over a short simulation, which guides the relaxation process towards a disentangled conformation. An illustration of the different types of links mentioned before as well as the definition of the virtual sites and repulsive energy terms for each kind of loop are contained in Supplementary Data Section 2.2 and Supplementary Figure S3 in detail. In the current pipeline, the piercing method is used to detect the links, while both piercing and Gauss methods are employed after the relaxation process to confirm the aptness of the model.

#### Tertiary contact search

SPQR allows the introduction of a harmonic potential energy on the $\mathcal {E}$RMSD between the simulated system and a reference structure (see Supplementary Data Section 2.3). This energy term can also be applied on arbitrary sets of nucleotides, and has been used for for enforcing secondary structure elements (34) or complex geometries as in intraviral RNA (35).

A short annealing simulation explores the conformational space around the unlinked, refined structure, in search for base pairs between nucleotides belonging to secondary structure elements which interact according to the Ernwin energy function. The geometry of the found contacts is enforced locally by a hard $\mathcal {E}$RMSD restraint (36), while the rest of the nucleotides are pushed towards the original structure with a softer restraint. Parameters are described in Supplementary Data Section 2.4. The result is a structure without clashes nor links; with the fulfillment of the possible tertiary contacts at a nucleotide resolution, and with a global structure as close as possible to the one proposed by Ernwin.

Finally, an atomistic model is obtained from the assembly of template nucleotides on each of the coarse-grained sites, taking into consideration the glycosidic bond angle and sugar pucker conformations. This step usually introduces minor artifacts such a broken covalent bonds, which can be easily removed by a short all-atom MD relaxation. The structure can be further refined by introducing an explicit solvent, which is explained in detail in Supplementary Data Section 2.5. The resulting structure is thus suitable for further calculations of SAXS profiles including solvent effects.

### Benchmark against solved PDB structures

To benchmark our pipeline against structures from the protein database, we used the reference structure’s exact coarse-grained pair distance distribution function (PDD) for the Ernwin potential and the reference structure’s secondary structure as basis for the RNA model. We chose the following 4 structures for our benchmark: 3R4F (66 nts), 4PQV (68 nts), 2R8S (159 nts) and 1L9A (128 nts) based on their length and the richness of their secondary structure. The selected structures do not have pseudo knots or large domains interacting with proteins. We then simulated 15 trajectories for each PDB structure with an energy function consisting of three components: The pair distance distribution energy, the A-Minor energy and the Loop-Loop interaction energy with different weights for each trajectory. As successful sampling with a strict separation between fragment library and benchmark structures have been shown previously (23) and we are now more interested the SAXS fitting and sampling efficiency, we now used a full fragment library from the whole representative set of RNA structures. We sampled 20 000–25 000 steps for each structure, which is more than should be necessary for small to medium sized RNA molecules. The simulations took between 12 and 36 h with one core used per trajectory.

We then selected two structure sets from each trajectory for refinement with SPQR: the structure with the lowest RMSD from the native structure, which reports the best structure Ernwin can produce, to test the ability of Ernwin to explore the conformational space, and the structure with the lowest pair distance distribution energy.

### Benchmark against real SAXS data

In contrast to proteins (37), no standard benchmark set of RNA molecules with known structures for which SAXS data are also available has been published. Hence, we searched the SASBDB (https://www.sasbdb.org) for SAXS data of RNA molecules which would work well to benchmark our pipeline. Luckily, at the time this paper was started, a recent deposit in this database, SASDK34, was ideal for this task: it contains a reasonably sized RNA molecule (118 nt) which is free of G-quadruplexes and overly complicated pseudoknots, and the corresponding paper (38) contained a predicted all-atom structure which we could use for comparison with our results.

For our benchmark, we took the NMR based secondary structure restraints from Supplementary Figure S7 of the paper we compare our results to (38) and added additional base pairs with RNAfold (39). This was achieved by modelling the NMR base-pairs as hard-constraints in RNA-fold (RNAfold -C), while leaving all other nucleotides unconstrained. The constraints and the final secondary structure can be found in the Supplementary Data Section 3.1. In contrast to other tertiary structure prediction programs, Ernwin does not predict additional base pairs, which made this step necessary.

We performed simulations using Ernwin with an energy contribution from the experimental pair distance distribution using 3 points per nucleotide in the Ernwin model and performed 32 simulations, half of which using simulated annealing and half with a constant temperature. Simulations used different weights for the energy function. The simulation lengths were set to 1500, 3000 or 5000 steps. We reconstructed a full-atom structure every 25 steps and calculated the CRYSOL 3.0 χ^2^ for all of the all-atom structures.

Later, we used SPQR to refine the structure with the best χ^2^ of each trajectory. For interpretation of the results, we assigned stacking to the helices by manual inspection of predicted structures.

### Evaluation of χ^2^

All reported χ^2^ values were calculated by CRYSOL 3.0 (6) over all-atom structures, which can be generated by the Ernwin reconstruction or a backmapping after SPQR refinement.

## Results

### Improvements to the Ernwin method

We could improve the efficiency of sampling and the quality of the predicted structures compared to previous versions of Ernwin: By replacing distance-only junction constraints with fragment based multiloop constraints, we strongly reduce the bias in the sampled junction topologies. This can be seen from the more structured (less uniform) distribution of angles between adjacent stems, even if they are only connected by the *broken multiloop segment*, as shown in Supplementary Figures S4 and S5 and Supplementary Data Section 3.2.

Additionally, we could improve the sampling efficiency and reduce the risk of getting trapped in a local minimum, by introducing new move types which change more than one fragment at a time. We can see that with the new move types, more unique multiloop conformations can be sampled in the same computation time (see Supplementary Tables S1 and S2 and Supplementary Data Section 3.3 for a direct comparison).

### Benchmark of our pipeline against published PDB structures

The benchmark of our pipeline against real PDB structures showed two main results:

- For the majority of structures, at least some trajectories converged towards a conformation with a low RMSD and good fit to the native structure.
- We also had at least one trajectory for each PDB structure that did not converge towards the global minimum but to an alternative conformation. These conformations often have a different arrangement of helices which lead to a similar overall shape. This explains why they are hardly distinguishable from the native conformation based on the pair distance distribution function alone. We will discuss this aspect in detail below.

These two results make it clear again that the strength of Ernwin is the exploration of the conformational space, while additional filtering of the results is needed to find the correct structure. When we use real SAXS data, we use CRYSOL for this filtering step. Table 1 characterizes the sampling trajectories in terms of RMSD to the native structure.

**Table 1.** RMSDs of predicted structures to the native PDB structure

	RMSD (Å) between the native structure and:				
PDB	Best PDD	Best RMSD	Candidate set		
	Structure	Structure	min	avg	max
1L9A	8.299	5.41	7.24	16.29	36.35
2R8S	15.782	15.198	15.78	25.80	32.12
3R4F	7.439	3.975	5.87	9.08	13.77
4PQV	13.391	8.387	8.39	12.75	17.26

The first column shows the RMSD of the structure with the best PDD, while the second column shows the best RMSD in the whole ensemble. The last three columns describe the candidate set, which is constructed by taking the best PDD structure of each trajectory. By using one structure per trajectory, we guarantee that they are completely independent of each other. Here, we give the best, average and worst RMSD in this candidate set. This shows that by constructing such a candidate set, we usually have at least one structure with an RMSD similar to the best overall RMSD in this small pool of candidate structures.

**Figure 3.**
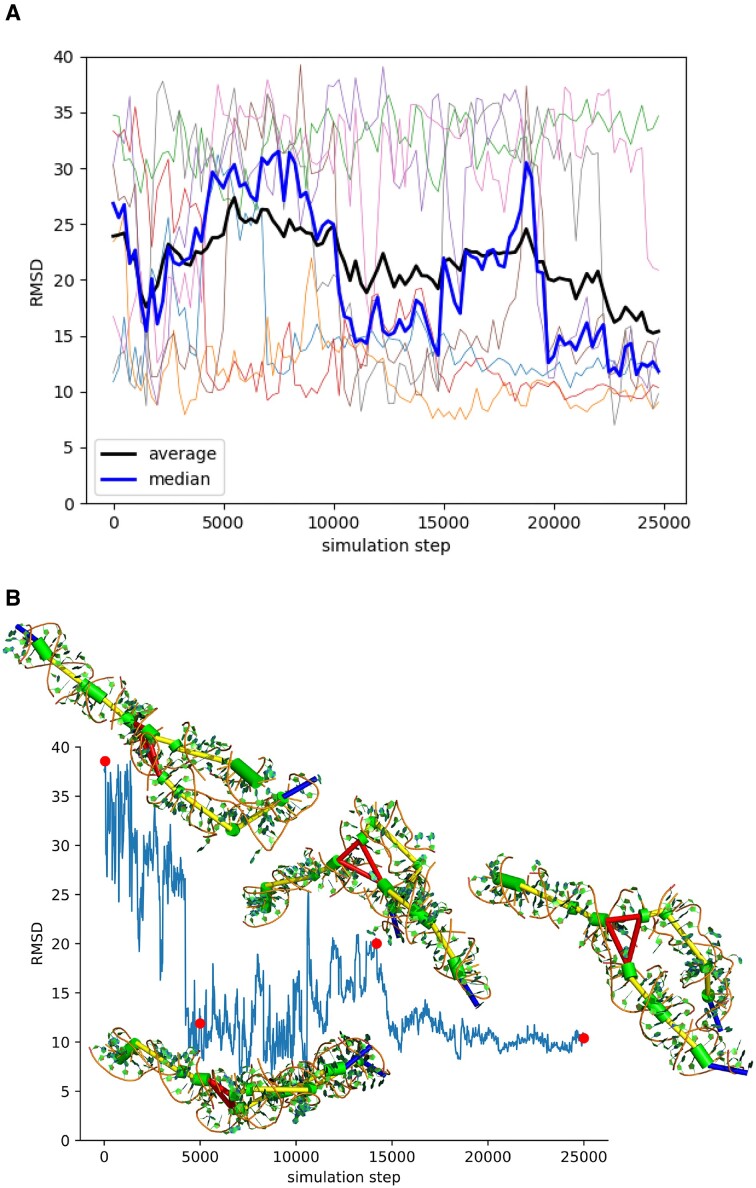
(**A**) The RMSD over the course of 8 trajectories, plus the median and average RMSD. (**B**) A successful trajectory, with some of the sampled conformations.

Structure 1L9A is an example of a structure where Ernwin performs very well: It has a length of 128 nts, is highly aspherical, making the SAXS profile and pair distance distribution function of the structure very distinctive, and it has a single multiloop with only three arms which could potentially clash. The median and average RMSD after the last step of the Ernwin simulation were 11.9 and 15.4 Å, which is very good considering the length of the structure. The best scoring (lowest PDD energy) structure of all trajectories had an RMSD of 7 Å. These results show how well Ernwin really can perform under good conditions. Figure 3, panel A shows a plot of the RMSD against the sampling step for all individual trajectories, whereas panel B shows one sampling trajectory for this structure in more detail.

Due to its length and secondary structure, 2R8S is the most challenging of the 4 structures we took for our benchmark. The predicted structure with the best PDD energy has an RMSD of 15.78 Å, which is also very close to the best RMSD overall found in the ensemble. This structure shows a good overall agreement of the global shape and conformation, but noticeable differences in the positions of individual nucleotides, as can be seen in Supplementary Figure S6. As with most RNAs, we again experienced some trajectories that got trapped in local minima with a reasonable PDD energy but large RMSD and overall shape. For the characteristic interior loop that introduces a 180° turn (*i1*), we have 43 fragments in our fragment library, and the correct fragment is recovered in 6.6% of all structures over all trajectories and is also present in the best PDD structure.

For 3R4F our benchmark shows lower RMSDs than for the comparable 4PQV structure, because of the distinctive bulge with five unpaired nucleotides. For this bulge, only four fragments were available in our library, one of which was the correct fragment from 3R4F. This example shows how the knowledge of a single fragment, e.g. via homologies or motif search, could improve the overall predictions, and how Ernwin could use homologies to its advantage. The predicted structure with the best PDD energy has an RMSD of 7.4 Å to the native structure and is shown in Supplementary Figure S7. One the other hand, 4PQV has no distinctive loops and none of the best PDD structures has fragments from 4PQV used for any interior or multi loop. See Supplementary Figure S8 for an example of a sampled conformation.

The duration of an Ernwin simulation depends on the complexity of the secondary structure (e.g. presence of complex multiloops), on the number of simulation steps and on the frequency of all-atom reconstructions (which are pretty slow). While good results are often available after an hour, we typically let our simulations run overnight or over the weekend to cover more simulation steps.

SPQR refinement results are summarized in Table 2. A large number of samples present links or piercings which can be detected and fixed by the methods presented here. The set of 1L9A contains a structure with a link between a three-way junction and an internal loop, which can be detected and removed after a minor redefinition of parameters, detailed in Supplementary Data Section 4.1 and Supplementary Figure S9. 2R8S presents the largest number of links, given its size and compactness. An example of two stems linked is shown in Supplementary Figure S10. From 10 hairpin-stem links detected in the whole pool of structures, 4 of them are involved in Ernwin contacts, which stresses the importance of the refinement for assessing the reliability of the structure. 3R4F is much less compact, and therefore, less topological artifacts are present, although there are links between two stems connected by an unstructured domain (illustrated in Supplementary Figure S11) and unable to form contacts according to the Ernwin energy function. In 4PQV, the links are frequently found between stems which are connected by an unstructured domain (Supplementary Figure S12). Analysis of the RMSD between the refined and unrefined structures shows that the presence and removal of links does not produce a substantial deformation of the structure. Moreover, the difference in this quantity can be attributed mainly to the clashes and the optimization of flexible loops during the refinement process.

**Table 2.** Analysis of refinement

PDB	*N*_Gauss_	*N*_Pierce_	*C*_Ernwin_	*C*_SPQR_	RMSD_N_	RMSD_L_
					(Å)	(Å)
1L9A	7	12	95	26	3.3 ± 1.4	3 ±0.9
2R8S	15	23	128	61	4.2±1.5	4±1.5
3R4F	2	7	0	0	1.8±0.1	2±0.1
4PQV	5	16	13	5	2.2±0.8	2.4±0.7

The set of structures for each PDB has 30 structures. The number of links in the set is denoted by NGauss and NPierce, detected using the Gauss integral and piercing methods, respectively. The number of tertiary contacts found in Ernwin is CErnwin, while CSPQR is this number in SPQR representation after refinement. The average RMSD of structures that were not initially entangled is calculated between the unrefined and refined structures in SPQR format and denoted by RMSDN. RMSDL contains only linked structures. Averages are reported with standard deviation.

According to Table 2, the number of contacts predicted by Ernwin and found in the exploration of SPQR is between 27% and 48%, excluding 3R4F. Nevertheless, in 1L9A one of the A-minors present in the native structure is successfully recovered in 3 instances of the 6 where Ernwin predicts it (see Supplementary Figure S13), while for 2R8S, no native A-minors are observed. Further contact analysis of the structures shows that the contact search greatly improves when the sugar pucker and glycosidic bond angle states are allowed to change. In fact, from a set of 150 structures selected randomly from the trajectories of 2R8S and 4PQV, the fixation of the pucker and glycosidic bond angle reduces the number of tertiary contacts found from 76 to 56 and from 183 to 97, respectively.

Finally, we see from Table 2 that the refinement procedure does not introduce a large deviation in the global structure. Visual inspection suggests that the highest RMSD differences correspond to the arrangement of clashed loops in general. Moreover, when introducing the all-atom details, the average clashscore decreases from 115.5 to 5.9 for the four structures described here (details are presented in Supplementary Table S3 in the Supplementary Data Section 4.2). This opens the way for a further analysis of the SAXS data with a full description of the solvent using more sophisticated approaches which are beyond the scope of the present work. A typical refinement run in SPQR takes from 1.2 to 3.7 min in the structures analyzed in this section, without links, and 1.5 to 5.3 in their presence, in both cases using a single processor of a desktop computer.

### Benchmark of our pipeline against real SAXS data

We also benchmark our pipeline against real SAXS data for the *Plasmodium falceparum* signal recognition particle ALU RNA, which was deposited in the SASBDB database under the id SASDK34. Soni *et al.* (38) predicted ab initio structures using the FARFAR webserver, selected the best cluster of structures based on scoring with CRYSOL (χ^2^ = 4.18) and then fitted the structures to the experimental SAXS data using SREFLEX (40) reaching a χ^2^ of 2.0 for their best model.

In our benchmark 14 out of 32 trajectories for the RNAfold secondary structure reached a χ^2^ <2 at least somewhere in the simulation and 4 trajectories had a χ^2^ <2 at the end of the simulation. We refined the best structure of each simulation with SPQR.

**Figure 4.**
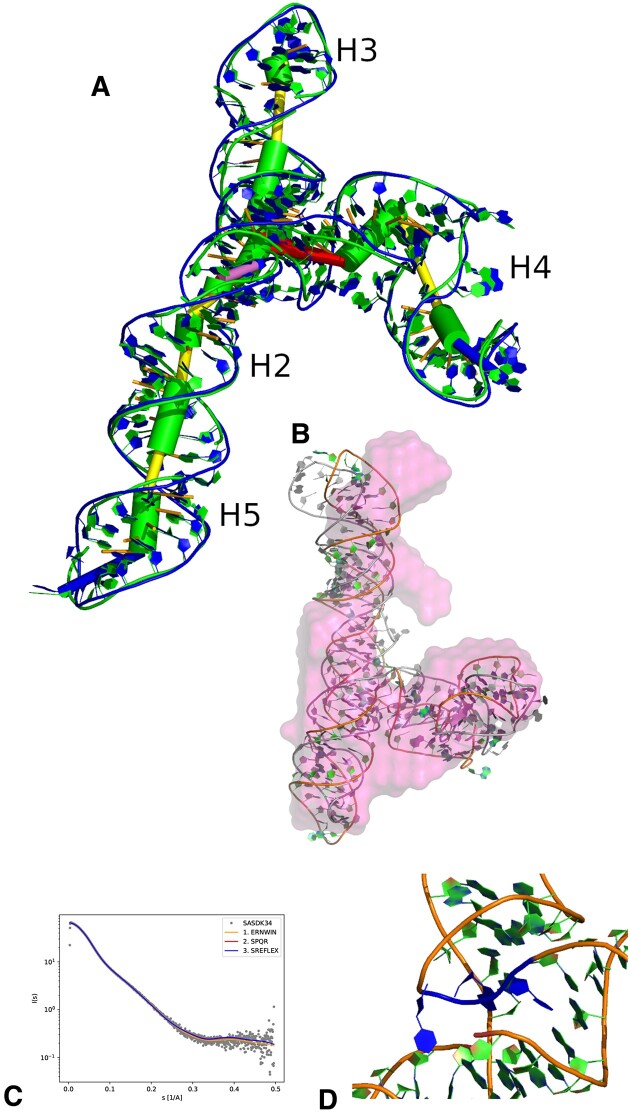
(**A**) Structure with the best χ^2^ for SASDK34. Green is the original Ernwin prediction and blue is the structure after refinement. (**B**) The best two independent predictions with final χ^2^ below 1.6 show a similar conformation with an RMSD of 7.7 Å to each other. In the image, they are both aligned to the volume calculated by Soni *et al.* (38) (not to each other) using SUPALM via the SASpy Pymol plugin (41). The best prediction is shown in color and the second best is shown in gray. (**C**) The predicted scattering curve for the best Ernwin prediction plotted in front of the experimental data from Soni *et al.* (38).(**D**) The UGU motif in our best prediction (colored in blue).

For most structures, the removal of clashes and links increased the χ^2^ slightly (see Table 3). At the end, 7 out of the original 14 structures still had a χ^2^ <2, five of which had a value <1.7 (see Figure 4 for an example). Further fitting the best refined structure to the SAXS data using SREFLEX (40) only slightly improved the χ^2^ from 1.492 to 1.481.

**Table 3.** Description of the structure with the lowest χ^2^ of the best trajectories

χ^2^ (Ernwin)	χ^2^ (refined)	Conformation
1.302	1.492	H3 and H2/5 collinear
1.473	1.844	unclear
1.537	1.690	H3 and H2/5 collinear
1.537	2.666	H4 and H2/5 collinear
1.580	2.134	H4 and H2/5 collinear
1.597	1.672	unclear
1.628	2.962	H3 and H2/5 collinear
1.699	1.924	H4 and H2/5 collinear
1.729	3.524	unclear
1.767	2.110	unclear
1.782	1.551	H3 and H2/5 collinear
1.834	2.976	H4 and H2/5 collinear
1.875	1.765	H3 and H2/5 collinear

The conformation was assigned by manual inspection. See Supplementary Table S4 in Supplementary Data Section S5 for an expanded version of this table that contains the values of the adjustable parameter.

Interestingly, the conformation of our best structure is different to the best prediction reported by Soni et al. (38). While their prediction shows the helices H3 and H4 as collinear, we find a better fit for stacking of helices H3 and H2/5, but still confirm the overall Y-like shape. In our best model the conserved UGU motif is at the beginning of a 180° turn which in our model is facing towards the helix H2, but due to the non-stretched conformation has ample space to fold out and for a potential interaction with a protein (see Figure 4D. As shown in Table 3, also other trajectories (and thus independent samples) favor conformations with the helix H2/5 in the stack. On the other hand, our best prediction with an architecture similar to the prediction of Soni et al. (38) has a χ^2^ of 3.0, and could be refined with SPQR and SREFLEX to a χ^2^ of 1.89.

Considering the near-equal length of the three arms and the fact that we are operating in a χ^2^ range that indicates a ‘not bad fit’ but does not indicate ‘the correct model’, it is not surprising that multiple different conformations and arrangements of the three arms can lead to good fits. Additionally, one must not forget the intrinsic flexibility of RNA, especially in such open conformations. The recorded SAXS data might arise from a dynamical ensemble, not a single structure. Thus such SAXS based all-atom predictions have to be combined with biological insights and additional experiments to find the correct solution.

The links found and removed with SPQR are less frequent in this case, given the lack of compactness. The few cases observed involved adjacent elements or elements connected by a short unstructured fragment, which did not exhibit tertiary interactions. From the pool of 32 structures, only 4 had links detected with the Gauss integral method and 10 with the piercing method. The average RMSD between the refined and unrefined structures was of 2.8 ± 0.9 Å and 2 ± 1Å, for the linked and not-linked models. The backmapping procedure makes use of steered-MD simulations minimizing the RMSD between the Ernwin prediction and the SPQR structure, as described in the Supplementary Data Section 2.5. On average, the clashscore was reduced from 101.7 to 0.9.

Finally, we used ARES (42) to score our best prediction as well as several predicted alternative conformations with low χ^2^ to assess the reliability of our models by other methods. ARES returned a predicted RMSD of 8.5 Å (after SREFLEX) for our best prediction as well as for our prediction that closest matches the structure from Soni et al. (38) (after Ernwin, with a χ^2^ of 3) and values between 6.5 Å and 10 Å for other alternative conformations. Note, however, that ARES was trained only on Rosetta-generated structures and it is unclear how well it performs for structures generated by other tools.

## Discussion

### Handling of junctions and ergodicity

Junction and pseudo-knot conformations are crucial for the overall conformation of the RNA molecule. In a system with fragments based on secondary structure elements, sampling junctions is quite challenging. For this reason, the authors in Laing *et al.* (43,44) have developed a random forest approach that classifies the junction family and assigns a geometry before sampling the rest of the structure with their tool RAGTOP (45). In contrast to this, it has always been an important feature of Ernwin to give junctions the flexibility to change during sampling. Here we successfully integrated this into the framework of a fragment based approach by assigning fragments to all junction segments.

The sampling of multiloops as individual segments in Ernwin requires the introduction of a constraint energy. Together with the clash energy, this causes certain conformations to have an infinite energy and therefore to be forbidden. This means that it is no longer clear whether the sampling is ergodic, as the forbidden regions of the conformational space might for some RNA secondary structures separate the conformational space into disjoint regions. We overcome this limitation by using new move types which exchange more than one fragment at a time, thus opening new paths around the forbidden zones of the conformational space.

### Challenges with estimating the pair distance distribution function

By using the pair distance distribution function instead of the scattering curve to guide our sampling, we avoid the problem of calculating the scattering curve from the predicted RNA structure at every sampling step, but depend on the quality of the PDD estimation from the scattering data. This conversion is implemented in many standard tools, such as GNOM (28) and these tools work very well in most situations. Still, there is the potential for (severe) errors during the conversion from *I*(*q*) to the PDD. First of all, care has to be taken that the conversion program is suitable for RNA molecule and does not use assumptions biased towards globular protein structures. Secondly, the transition from observed intensities to the pair distance distribution function depends on an estimate of the maximal interatomic distance in the unknown structure and a wrong value here can potentially cause hard to estimate errors in the pair distance distribution function. This problem can be dealt with by manually or automatically (46) calculating the PDD for different values of *D*_*max*_ and selecting the PDD curve with the best properties. Obviously defining these properties is not trivial.

Our benchmarks show that at least for the structure we used, errors in the PDD caused by a wrong *D*_*max*_ as input to GNOM can be compensated quite well during Ernwin’s sampling (see Supplementary Data Section 6, Supplementary Figures S14 and S15).

### Quality of the pair distance distribution energy for fitting towards SAXS data

The benchmarks have shown that the use of the PDD energy enables the model to sample structures in good agreement with experiment. Nevertheless we observe that robust predictions require one to perform multiple simulation runs to make sure the correct structure is among those that were generated. In addition, we recall that multiple structures might have degenerate PDD-energy, thus making it difficult to identify the correct one.

**Figure 5.**
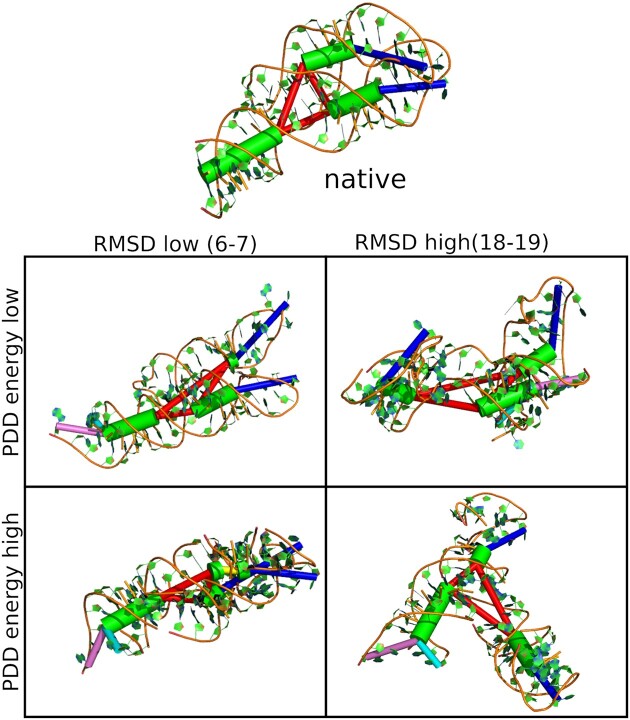
Sampled structures for 1Y26: It can be seen that the structure at the top right of the table (low PDD-energy, high RMSD) adopts a similar global shape as the native structure, but the arms of the junction are swapped. While the two hairpins are kissing in the native structure, they are at opposing ends in this high-RMSD structure. On the other hand, the structure at the lower left (low RMSD, high PDD energy) looks overall similar to the native structure, but is more compact than the native structure. Note that this figure shows structures before refinement with SPQR.

This situation is especially likely to happen for structures with several arms of nearly equal length, which exhibit a certain symmetry at the level of helix representation. For example, the PDB structure 1Y26 (which was used exclusively in the parametrization of Ernwin) represents a junction with three arms of similar length. Figure 5 shows the native structure, a sampled structure with low PDD energy, and low RMSD and a sampled structure which has a low PDD energy despite having a high RMSD. This case is a good example of structures where the loop-loop interaction energy of Ernwin helps favoring the correct structure more than the PDD energy does.

Still, these limitations of the energy function made it necessary to include a filtering step based on the χ^2^ score into our pipeline. On the other hand, the energy is good enough to reliably generate faithful structures in a significant percentage of the trajectories, thanks to the ability of Ernwin to quickly and efficiently explore the conformational space, although more accurate estimations of the PDD could be used in the future in order to improve the results.

### Why report a unique structure

Although Ernwin is able to use ensemble based energies, we have chosen to present a single ‘best’ structure for two reasons: First of all, individual structures as illustrative examples of the ensemble are commonly used in biology to simplify things. For instance, this is commonly done when reporting minimum-free-energy secondary structures for RNA. Secondly, it is hard to validate ensemble predictions. On the other hand, individual structures can be easily compared to high resolution crystal structures.

### SAXS and SPQR

We intentionally do not constrain the SPQR refinement with SAXS data. Ernwin alone does a good job at predicting structures which fit the experimental SAXS pattern well, because SAXS measures a global effect which does not depend on the local orientations of individual nucleotides, which will be refined with SPQR. On the other hand, SPQR does not greatly distort the structures and can turn some models into a more realistic fashion. We see that the good χ^2^ of our best prediction is retained after the refinement, while for some other predictions, the agreement with the SAXS data gets moderately worse. It would be interesting to see if this step of refinement and relaxation without SAXS constraints helps to filter out false positive predictions of Ernwin and to avoid overfitting, but this will be the subject of future investigations due to the lack of experimental structures with both SAXS and 3D structural data available at the moment.

## Conclusion

We introduced here a truly multiscale methodology to propose and reconstruct RNA structures up to 159 nucleotides subject to SAXS experimental restraints. Our approach shows how the conformational space can be explored efficiently using the assembly of secondary structure based fragments, which has been improved for sampling realistic multiloop conformations and for enhancing the exploration of the conformational space by using different types of moves.

By refining the structures with SPQR, it is possible to remove artifacts induced by the coarseness of Ernwin, which deal with the clashes, topology and the possibility of implementing the tertiary contacts at a nucleotide-level resolution. Moreover, this refinement can be extended to other fragment assembly methods, which are also affected by similar artifacts (31). The pipeline ends with the structures refined in all-atom representation in explicit solvent, which allows to analyze further effects of the buffer into the SAXS profile (10).

Overall, our multiscale method takes the best of each representation, and constitutes a powerful tool in the structure prediction problem of RNA macromolecules in solution.

## Supplementary Material

gkae602_Supplemental_File

## Data Availability

The code of Ernwin can be downloaded at https://github.com/ViennaRNA/ernwin. The code of SPQR can be downloaded at https://github.com/srnas/spqr. The best model proposed here for SASDK34 is included in the Supplementary Data, under different levels of refinement. Installation instructions and tutorials are included in both websites. Refined and unrefined structures, SAXS data calculated for our best model and source codes can also be found in https://doi.org/10.6084/m9.figshare.26078686.
